# Design and Experimental Characterization of L-CADEL v2, an Assistive Device for Elbow Motion

**DOI:** 10.3390/s21155149

**Published:** 2021-07-29

**Authors:** Marco Ceccarelli, Mykhailo Riabtsev, Axel Fort, Matteo Russo, Med Amine Laribi, Monica Urizar

**Affiliations:** 1 LARM2: Laboratory of Robot Mechatronics, University of Rome “Tor Vergata”, 00133 Rome, Italy; 2 Department of Mechanical Engineering, University of the Basque Country (UPV/EHU), E-48013 Bilbao, Spain; riabtsev.m@gmail.com (M.R.); monica.urizar@ehu.es (M.U.); 3 Département Génie Mécanique et Systèmes Complexes, Institut PPRIME, Université de Poitiers, 86073 Poitiers, France; axel.fort@etu.univ-poitiers.fr (A.F.); med.amine.laribi@univ-poitiers.fr (M.A.L.); 4 Faculty of Engineering, University of Nottingham, Nottingham NG8 1BB, UK; matteo.russo@nottingham.ac.uk

**Keywords:** assistive device, elbow motion, design, cable-driven parallel manipulator, rehabilitation robot, experimental analysis

## Abstract

An experimental characterization is presented for an improved version of a wearable assistive device for elbow motion. The design is revised with respect to requirements for elbow motion assistance, looking at applications both in rehabilitation therapies and exercising of elderly people. A laboratory prototype is built with lightweight, portable, easy-to-use features that are verified with test results, whose discussion is also provided as a characterization of operating performance.

## 1. Introduction

The elbow is a complex joint in which the distal end of the humerus meets the proximal ends of the radius and ulna. An elbow joint can perform flexion/extension motion, acting as a hinge, as well as enabling the rotation of the forearm around its axis in a pronation/supination motion. As one of the main joints of the human body, it plays a critical role in the quality of life, greatly impairing manipulation in the case of injuries. Most of the common diseases of the elbow are either due to a traumatic injury, such as a fracture or a dislocation [1] or to joint overuse, such as tennis elbow and golfer’s elbow [2]. Furthermore, arthritis and strokes can also significantly affect the elbow’s performance, and they must be more and more considered in elderly people.

Physiotherapy is fundamental for the management of elbow dysfunctions, with active treatments that aim at restoring full joint mobility after an injury currently performed by physiotherapists in one-to-one sessions with patients [3]. Nonsurgical rehabilitation programs include both stretching and strengthening exercises, which are performed with the active support of a physiotherapist in the early (acute) phase of the rehabilitation [4]. Similar to rehabilitation assistive devices, medical training systems can be developed for assistive motion exercises for elderly people. In principle, those assistive rehabilitation devices also can be used for exercising elderly people after some adaptation.

In the last decades, automated and robotic rehabilitation systems have been proposed for patients to carry out rehabilitation by themselves. By enabling rehabilitation at home and without in situ supervision of a physiotherapist, these systems result in an economic advantage, as well as in practical usage, since patients save commuting time and physiotherapists can focus on critical cases and diagnosis rather than routine training activity [5].

Early examples of robotic rehabilitation systems that managed to automate elbow injury treatment can be found in the early 2000s. Several pioneering works discuss robot-aided rehabilitation for stroke patients as in [6,7,8]. The rehabilitation robot FRIEND, developed at the University of Bremen, supports patients with upper limb impairments with a wheelchair-mounted manipulator [9], similar to the more recent solution in BIT wheelchair [10]. While these early systems have been successful in automating the rehabilitation process, their size and price make them unsuited for home rehabilitation since they were designed to be deployed in clinical settings.

For this reason, wearable rehabilitation systems have been developed in recent years. In [11,12], for example, wearable exoskeletons are proposed for rehabilitation purposes. However, despite the system mobility, a significant encumbrance of their bases makes them difficult to transport and use at home. Lighter design concepts presented in [13,14,15,16,17,18,19] offer solutions that can be easily stored in a relatively small space but still require a support frame for the rehabilitation exercises.

Fully portable devices that can be worn and used without any support frame are characterized by more challenging requirements since they need to exert highly controlled forces on patients while being lightweight with a compact design shape. In [20], an SMA-based wearable device is introduced. However, cable-driven mechanisms are preferred due to their inherent safety in case of system failure, as pointed out in [21,22]. In [23,24], examples of cable-driven wearable rehabilitation devices are reported with systems that can perform the full rehabilitation exercise without requiring any external frame or support and are easy to transport so that they can be easily used at home for self-exercising.

In this paper, the evolution of the CADEL device, a cable-driven elbow rehabilitation device whose preliminary work is introduced in [25,26,27,28], is reported with a focus on a novel low-cost prototype design and its experimental characterization. The paper is organized as follows: Section 2 presents the requirements that have been considered in developing the improvement to the original design of CADEL and its different versions in order to build a prototype for practical implementation. Section 3 discusses the solutions for mechanical design and mechatronic components up to the present construction of a prototype, with market components and 3D printing manufactured parts; Section 4 is focused on the experimental validation of the prototype design, with a performance characterization of the operational features through test results; and the Conclusions summarize the contribution of the paper.

## 2. Requirements

Robots have been steadily gaining importance, especially in industrial applications, but also entering other fields such as medicine, entertainment, construction, space, or personal use, such as the examples in [29,30]. Those robots performing useful tasks for humans, or equipment excluding industrial applications, fall into a different group, the so-called service robots [30]. The role of robotics in movement disorders and post-stroke patients’ rehabilitation has been investigated intensively, as pointed out in [31]. In fact, studies have highlighted a need to provide rehabilitation procedures with some key features for the effectiveness of repetitive performance and a rehabilitation strategy. It is also important that a patient can perform an assigned task continuously and without dependence on the physical presence of an assistant. Similarly, the exercising of elderly people is addressed with tasks that need to be run in efficient continuous operation, very likely without any assistance from other operators.

For these reasons, the use of robotic platforms for rehabilitation is of particular importance, as pointed out in [5]. Robotic rehabilitation is an effective strategy for at least partial recovery of the lost functions, and robot devices are good solutions for home exercises of elderly people too. Robot medical devices allow precise and repeatable movements that can improve motion capabilities by activating critical muscles during the exercises. The repetition of the same gesture leads to improvement in function recovery, provided that the use of the robotic technique is frequent and prolonged. Robotic rehabilitation/exercising features many benefits, such as repeatability, autonomy during task execution, the chance of studying and validating new protocols based on new motion laws, and the introduction of different strategies to grant faster and better recovery, as pointed out in [32]. Table 1 presents a list of significant examples of existing rehabilitation/exercising devices with robot structures with cables. For each device, the number of DoFs (degree of freedoms), joints, actuation type, as well as power transmission are given as an indication of main characteristics.

**Figure 1:**
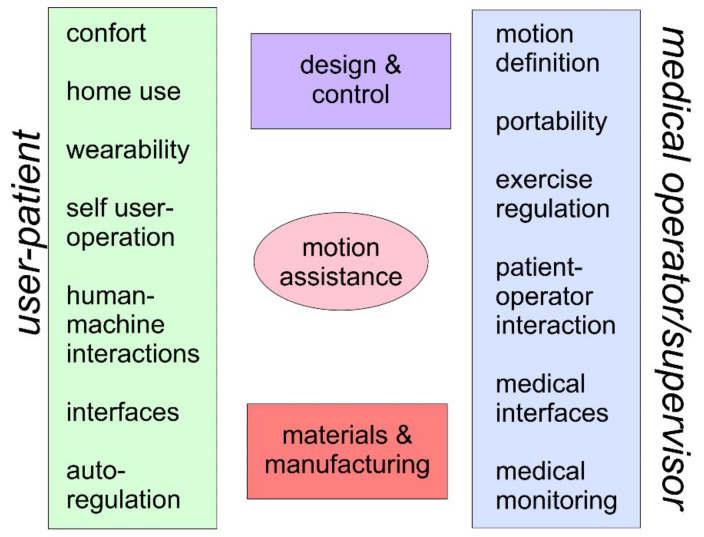
Open issues on motion assistance of the elbow articulation.

Open issues related to motion assistance of the elbow articulation can be recognized as a specific application in arm physiotherapy. With reference to Figure 1, aspects relating to interaction with patients and interaction with medical operators can be recognized as fundamental. These aspects may have an important impact not only in a concrete application of motion assistance but even in the design of the structure and in the regulation of its functionality, as well as in the choice of materials and production processes that can lead to suitable devices for both physiotherapy needs and for the satisfaction of patient users. In particular, the following aspects are emphasized in Figure 1, as differentiated but shared from the point of view of patients and medical operators.

As per the patient side, reference can be made mainly to:

- **Comfort**: User comfort can be a requirement that gives constraints that are often not considered for the functional medical purposes of a motion assistance device. The comfort in wearing and utilizing the device requires considerations not only in design and functional problems but also in man-machine interactions and the psychological effects on the user.
- **Home use**: The solution of a portable device that can be used at home is a problem both for the design and for the operation of a motion assistive device that must be managed directly by a user in an environment, such as the home environment, that is not designed for medical devices. Therefore, it may require particular attention both in terms of sizing and functionality, as well as interactivity with a user, also bearing in mind additional requirements on medical and environmental health and hygiene.
- **Wearability**: Wearability is linked both to comfort and to the possibility of home use. It is also an important aspect for user acceptance, requiring not only comfort and transportability but also aspects of interaction with the patient’s arm and the ease of application and use of the device. These problems certainly influence the design and operation of the device, also requesting specific sensors to define an optimal solution.
- **Self-user operation**: Autonomy in managing the device for motion assistance is a fundamental aspect for determining a device that can be managed directly by a patient, considering both learning and handling the functionality of the system, as well as its regulation and monitoring during motion exercise. This aspect also includes device quality in terms of training and ease of device management.
- **Human-machine interactions**: The interactions between the limb and the device determine problems and characteristics of the solution for motion assistance that can be understood, not only as requirements and constraints, but also as indications for the design and functionality of the device at adequate levels, both in terms of movements and actions between the device and the user’s limb.
- **Interfaces**: The interfaces that can be required for system operation are not only at the level of movement adjustment for the exercise by the user but also for motion and clinical monitoring. Interfaces that can facilitate the understanding of the device by a user and its management, such as the supervision of the system via smartphone, are desirable.
- **Auto-regulation**: The regulation and, therefore, the control of the functionality of the device in the exercise of motion assistance requires supervision algorithms that can be independent of the user’s reaction. Indeed, it is required that the device can have self-regulation to ensure a certain level of autonomous operation for the user’s personal safety.
- **Safety**: User safety is an essential aspect in these motion assistive devices, where a machine interacts with the human body. Thus, the need to ensure high-reliability levels imposes design choices and controlled functional procedures in every aspect of risk, which, however, must also be shared with risk awareness by users and medical operators.

As per the medical operator side, reference can be made to:

- **Motion definition**: The definition of the motion characteristics in the exercise of motor assistance requires clinical medical knowledge of a patient by a medical operator and the possibility of applying this exercise adequately with the device available. The definition of motion exercise requires synergy and integration of the device’s motion skills with the physiotherapy requests that are identified by a medical operator specifically for a user-patient.
- **Portability**: Portability is a specific requirement of these motion assistive devices that make them very suitable to the needs of patients and to the possibility for physiotherapists to adapt motion assistance with specific patient requirements. This portability feature, however, requires considerable attention in terms of lightness in the device, as well as its easy functionality, easy adaptability to patient anatomy, and operation management by a patient himself with few elements supporting the device.
- **Exercise regulation**: Exercise regulation is a very important aspect of motion assistance that must be manageable both by a user and by a medical operator supervising a medical therapy. Therefore, regulation and control of the movement exercise and of the sensors that are connected to it must be accessible both for a patient and for monitoring and remote control by a medical supervisor. Therefore, both autonomy and control open to contextual and temporary needs of the patient and medical supervisor are required.
- **Patient-operator interaction**: A way of interacting between the patient and medical operator during the motion-assisted exercise must be provided so that they can both react to the real-time reactions to the exercise and make necessary adjustments, as well as obtain data on the achieved results.
- **Medical interfaces**: Medical interfaces are indispensable aids for a motion assistance device with regard to clinical and medical parameters during and in response to motion exercise. Such medical interfaces, in terms of both sensors and systems, as well as a visualization interaction, can be useful complements, and they must be integrated into the structure and functionality of the device.
- **Medical monitoring**: Medical monitoring can be considered indispensable not only for the supervision and evaluation of the effectiveness of the motion exercise but also for the possibility of giving timely indications to a patient-user during and in anticipation of an exercise. Medical monitoring systems can be composed of the same sensors as the medical interfaces but can also include special devices that can allow remote monitoring with video transmission.
- **Safety**: Aspects of safety, including medical ones, are certainly to be considered as a priority in the applications of devices for motion assistance, and from a medical point of view, they also require non-technical considerations that can continuously identify new problems and new solutions in terms of both technical and medical devices.

The above-mentioned aspects can also be considered as an overview of the open issues and the related problems that should be addressed in the development of a technical-medical device for motion assistance in rehabilitation, mobility, or training exercises. The aspects considered above, as well as others that can be identified on the basis of specific applications, are the bases for both technical and medical considerations that must be taken into account separately and in integrated ways in the design and functionality of a system for motion assistance. Only in this way will the system be satisfactory for the three levels of use, namely, patient, technical side, and medical application.

## 3. Conceptual Design

**Figure 2:**
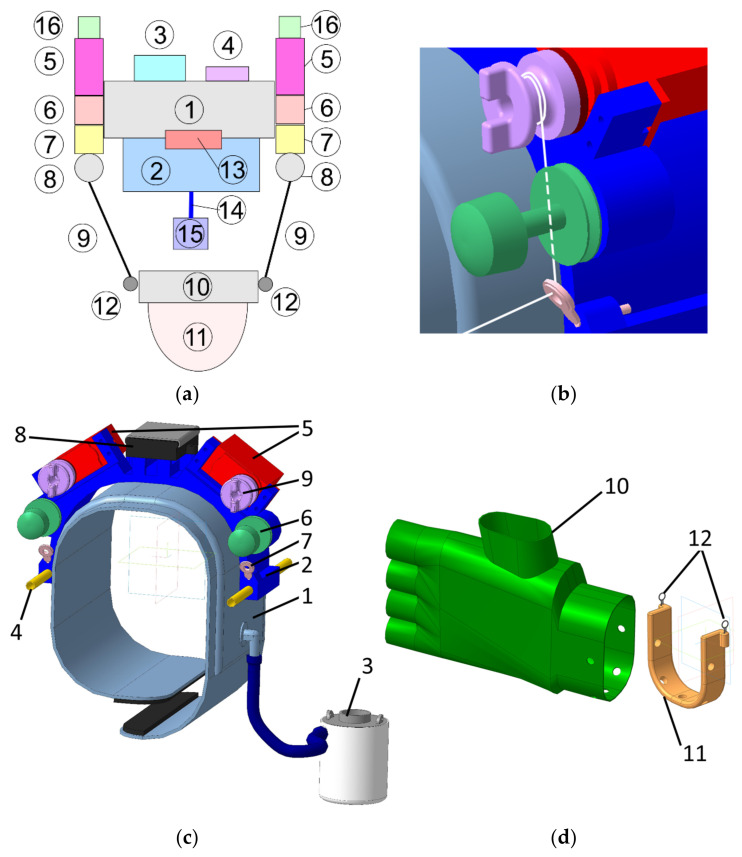
A design solution for the mechanical design L-CADEL v2: (**a**) A conceptual box diagram; (**b**) A scheme of a design solution for cable tensioning; (**c**) Upper module; (**d**) Lower module.

The proposed design of L-CADEL v2 is presented in Figure 2, with an upper module and a lower module. Figure 2a shows a conceptual design with a box diagram, from Patent 5, whereas Figure 2c shows a mechanical design with all the components for the upper module, wearable on the arm near the shoulder, and Figure 2d shows the structure of the lower module, wearable at the wrist zone of the forearm. The structure of the upper module corresponds to a fixed platform and the lower module to the mobile platform of a cable-driven parallel manipulator.

The upper module contains two main parts: an inflatable cuff (1) and a lightweight plastic arc-shaped platform (2), to which the actuators and other components are attached. The inflatable cuff is taken from a conventional manual blood pressure monitor. In this design, it serves as an interface that fixes the device on a person’s arm and secures it from any kind of motion by inflating the cuff with the pump (3). The cuff is connected to the arc-shaped platform with a hook-and-loop fastener and two strings (not shown in Figure 2) that are put through the aluminum tubes (4), which are at the extremity of the arc-shaped platform. The arc-shaped platform serves as a base part, where the actuators (5), tensioners (6), guiding rings (7), power source (8), Arduino Nano, and DC-DC step-up converter are fixed. The actuators are continuous rotation servomotors. On the shaft of each servomotor, a pulley (9) for the cable is fixed.

The lower module consists of a glove (10) and a lightweight plastic arc-shaped platform (11). These two main parts are attached to each other with screws. The cables are attached to the small rings (12) made of metal wires that are fixed to the platform.

**Figure 3:**
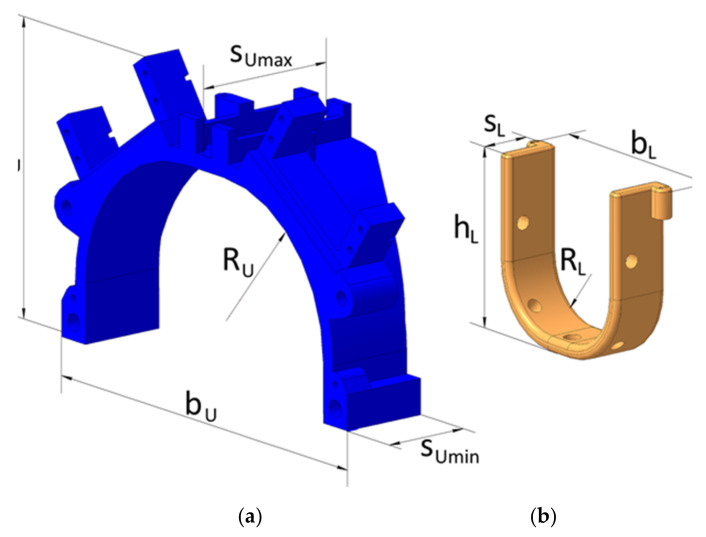
Main design parameters of L-CADEL v2 platforms in Figure 2: (**a**) upper module; (**b**) lower module.

The cable driving units can be considered as a system with a motorized reel, which pulls the anchor point. These systems have low friction, which allows the reduction of the power and size of the actuators. The cable drive design is presented in Figure 2b. The pulley has two ledges, which provide easy manual adjustment of the cable length and tension. The tensioner is necessary to preserve the tension in the cable between the pulley and the tensioner, and it also prevents the cable from tangling and going off the pulley. A conventional tensioner for sewing machines is used in this design solution. It consists of an adjustment screw, a spring, and two polished washers, in between which the cable passes. After the tensioner, the cable goes through a guide ring towards the lower module. The guide ring is a commercial fishing rod guide ring, which has a ceramic core for friction reduction. As it can be noted from Figure 2b, the cable passage is straight inside the tensioner, being bent only in the guide ring, with the aim to reduce the friction between the cable and the cable transmission elements. The mechanical design of the main platform structures is shown in Figure 3 for the upper and lower modules with the main parameters for sizing a prototype.

**Figure 4:**
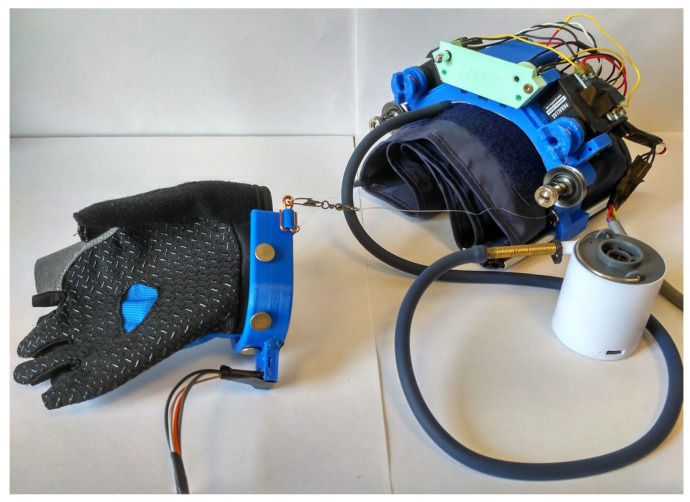
A prototype of L-CADELv2 with the lower and upper modules built at LARM2 in Rome.

**Figure 5:**
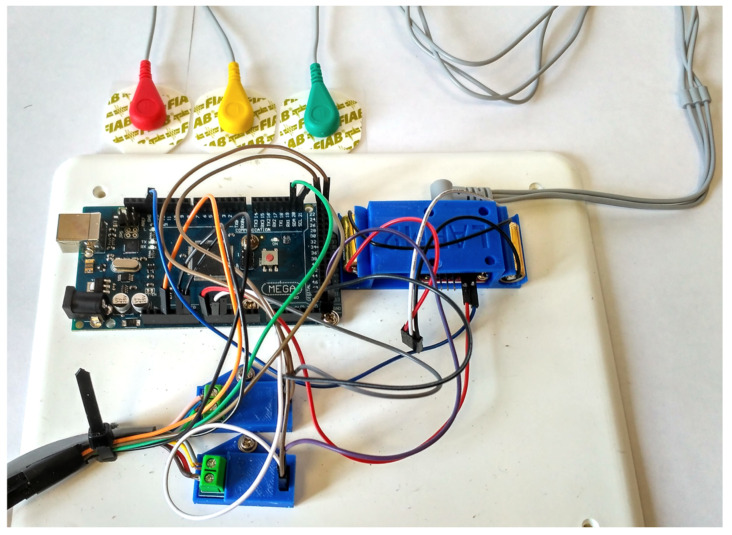
The data acquisition system of the prototype in Figure 4.

A prototype of the proposed L-CADEL v2 device has been built at the LARM2 laboratory in Rome, as shown in Figure 4, with the dimensions listed in Table 2. The platform parts of both modules are 3D printed using fused deposition modeling (FDM) technology. Parallax continuous rotation servos [42], providing a maximum torque of 27 Ncm, are used as actuators, controlled with an Arduino Nano [43], and powered by three AAA batteries with step-up DC-DC converters to provide 5.5 V. The Arduino board is equipped with a buzzer to indicate start, stop, and low battery charge. In order to assess the prototype performance, the lower module is equipped with an inertial measurement unit (IMU) [44]. In addition, the current consumption of the upper module servomotors is measured with two current sensors, and the muscle activity of a user is assessed with EMG sensors [45]. The sensors are connected to a separate Arduino Mega [46], as shown in Figure 5, in order to decrease the electrical noise that can be generated by the servomotors.

## 4. Results and Discussion

**Figure 6:**
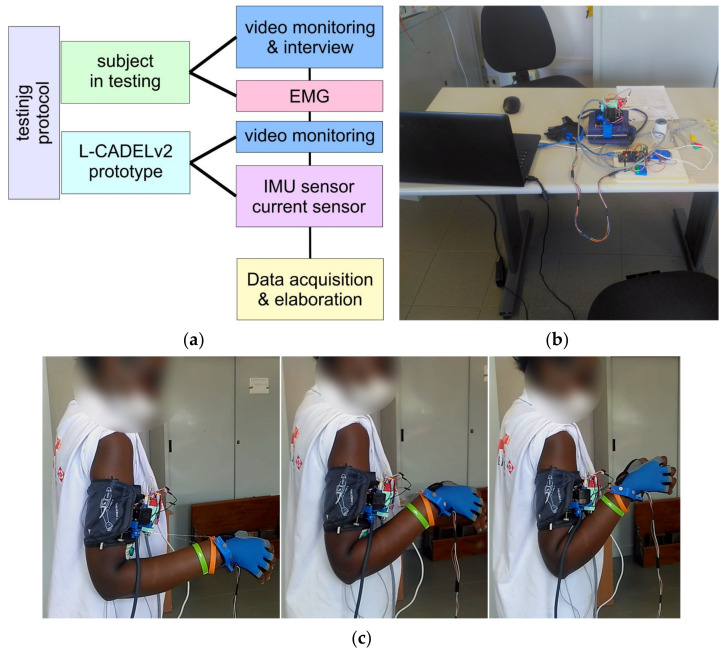
Setup for testing L-CADELv2 prototype: (**a**) A conceptual scheme; (**b**) Lab setup at LARM2 in Rome; (**c**) Snapshots from a lab test.

**Figure 7:**
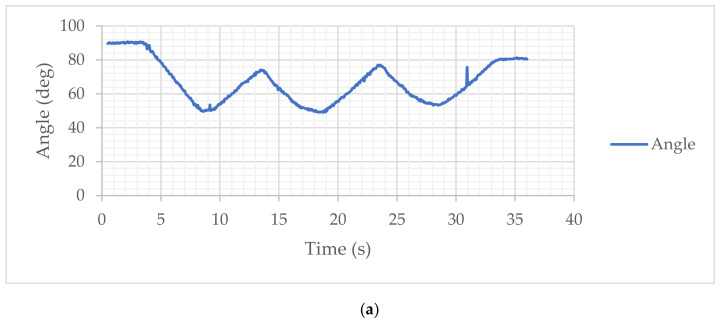

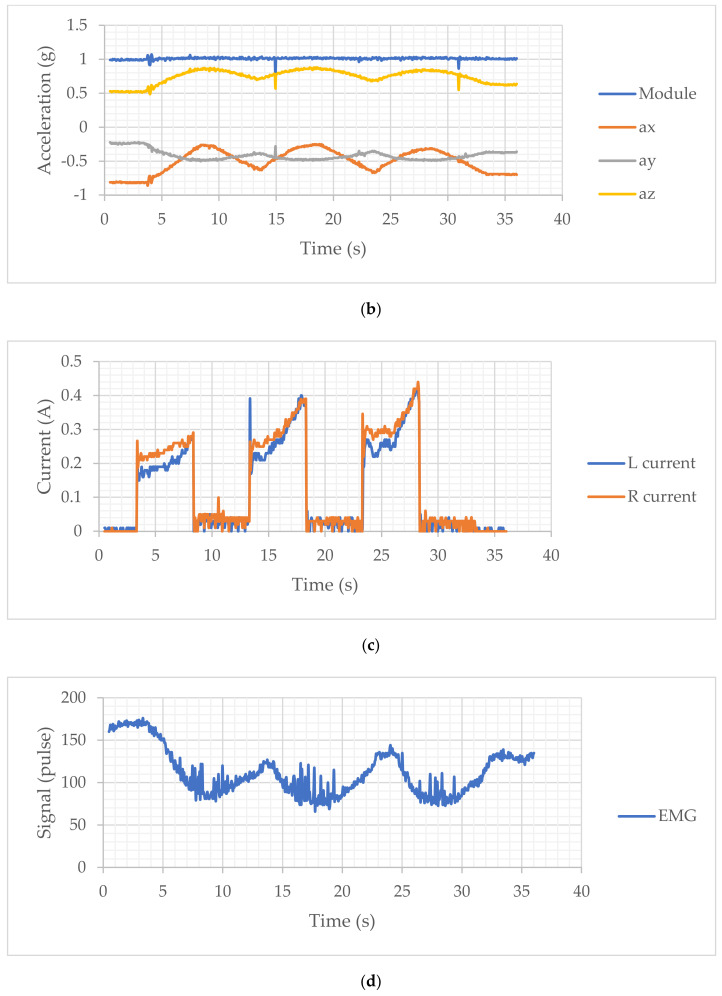
Results of a free-load assisted motion: (**a**) Acquired elbow articulation angle; (**b**) Acquired acceleration of wrist; (**c**) Acquired power consumption (from drawn motor current); (**d**) Acquired EMG sensor signal.

**Figure 8:**
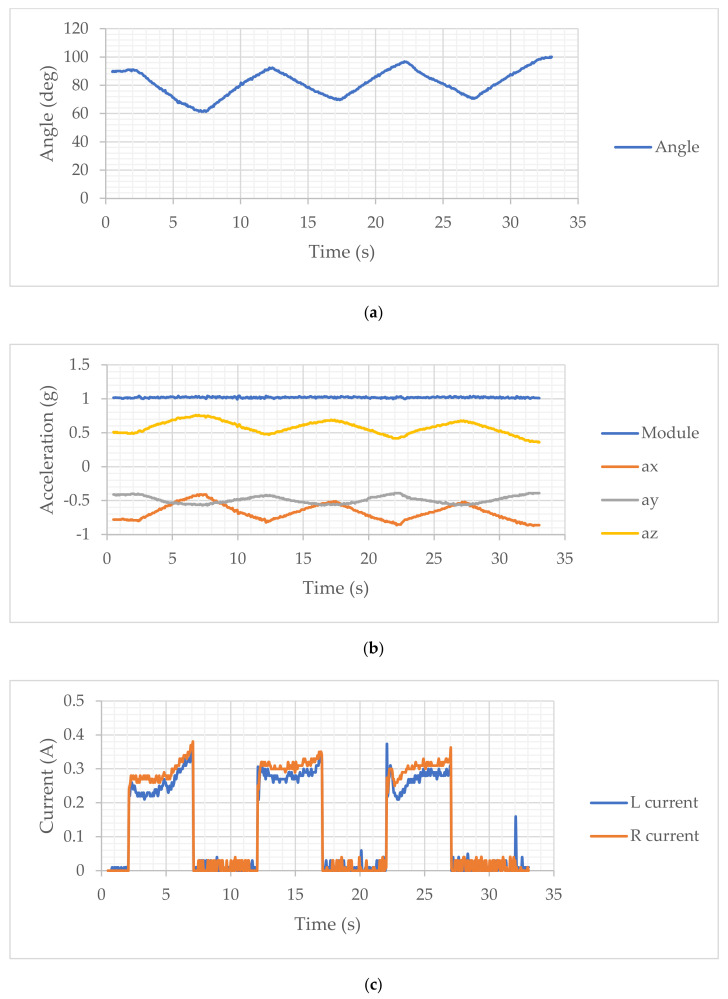

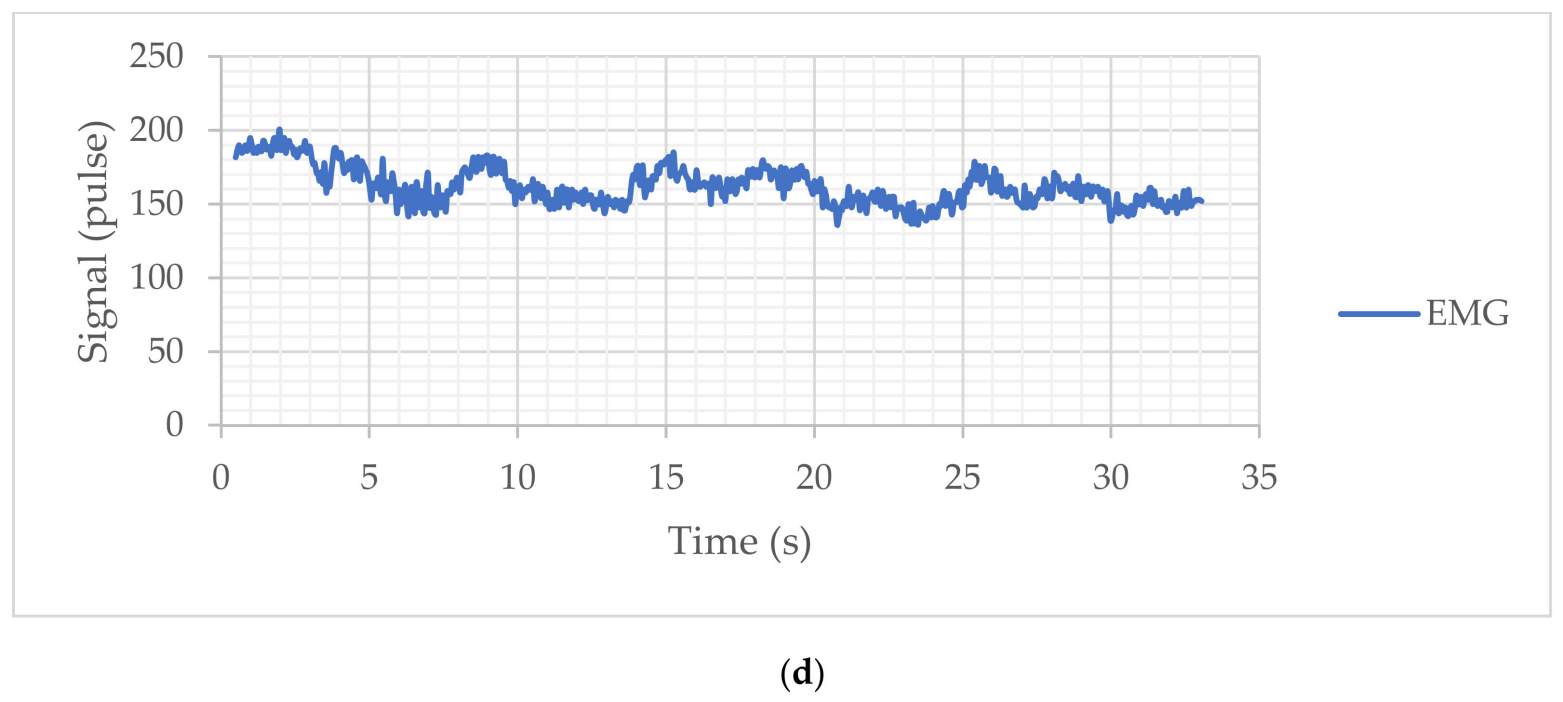
Results of an assisted motion with a 5 N load: (**a**) Acquired elbow articulation angle; (**b**) Acquired acceleration of wrist; (**c**) Acquired power consumption (from drawn motor current); (**d**) Acquired EMG sensor signal.

The L-CADEL v2 design has been validated with experimental tests at the LARM2 laboratory of the University of Rome “Tor Vergata”. The setup has been organized as outlined in the conceptual scheme in Figure 6a and implemented as shown in the picture in Figure 6b. An experimental validation campaign was carried out using the prototype according to an appropriately designed protocol (see Appendix A) with three volunteers aged between 20 and 25, who were selected among the students attending the LARM2 laboratory in Rome. The experimental testing activity was carried out with reference to two modes of use of the prototype, i.e., one with an unloaded, completely passive arm, and another test mode with a reactive arm loaded with a weight of 5 N. The validation results are shown in Figure 7 and Figure 8 and Table 3, as an experimental characterization of the prototype for the possibility of implementation in a medical/clinical setting. The modalities of the test have been designed according to the protocol scheme in Appendix A, with a well-defined sequence of steps, the prototype that is equipped with its sensors, and an EMG sensor that is applied on the arm muscle to evaluate the muscle reactivity of the arm under experimentation. In addition, visual monitoring of the operation is activated to verify the activity of the subject with the possibility of recording for subsequent evaluations, as in a clinical-medical implementation. The snapshots of Figure 6c show an example of this testing method in the LARM2 laboratory and the related visual monitoring. The assisted movement of a test consists of three flexion-extension cycles with a natural speed for the subject at a frequency of approximately 10 s per cycle, and it is performed with three repetitions to have a sufficiently statistically significant response. The test is carried out with the arm completely unloaded or with a weight of 5 N (using a small bottle of water grasped by the hand) by asking the subject to hold it with the muscles inactive.

In particular, the results reported in the monitoring of the movement through the angles and accelerations acquired by the IMU allow the highlighting of the following characteristics. In Figure 7a and Figure 8a, a regular movement can be noted for the motion assistance in the extension and flexion phases, with a small tremor probably due to the anatomy of the human arm and to the flexibility of the device fixing its two modules on the user’s arm. The range of motion in the unloaded arm test is approximately 25 deg, and it is slightly widened in the loaded arm mode due to the fact that the load aids in the extension of the arm.

In Figure 7b and Figure 8b, the acquired acceleration is reported with its Cartesian components. The actual acceleration of the exercising motion must be considered, including the gravity, which determines the modulus at an approximately unitary value with an almost constant trend. The movement components show a very limited variation, which is a consequence of the regularity and smoothness of the assisted movement coming from a regular operation of the servo actuators that implement the relative movement between the two modules. In particular, it can be noted that the x and y components show a similar and congruent trend with the angular motion with very limited variations. However, the accelerations acquired in the case of an unloaded arm are comparable with those acquired in the case of a loaded arm, indicating the efficiency of the device in being able to manage the assisted movement similarly in both modes.

The plots in Figure 7c and Figure 8c report the actuation of the actuators with a current intensity of about 0.3 A. These values indicate a differentiation between the two actuators since, despite the movement of the arm occurring in the sagittal plane, the two actuators and the corresponding cables can be found not to be perfectly parallel and aligned with the sagittal plane. These results, among other things, are also influenced by the effect of gravity. However, it seems that in both modes, the actuators show an increase in actuation near the end of the cycle, probably also linked to the configuration of the arm in the closing configuration in the flexion phase. The effectiveness of the movement on the arm is evaluated using the response of the EMG sensor mounted on the arm muscle. The acquisitions are reported in Figure 7d and Figure 8d, which show a differentiated behavior as a function of the presence of the load on the arm. In the case of an unloaded arm, the muscle responds by following the assisted movement of the arm, even with small signals of activation of the muscles that may be in an involuntary form, which cannot be precisely defined. In the case of Figure 8d, the response of the EMG signal indicates an almost constant muscle activation, probably due to the fact that the load on the arm requires an action of the muscle that the subject has also activated instinctively. Nevertheless, it can still be noted that the device determines an influencing action on the muscular activity of the arm.

In conclusion, the results of the validation tests are represented by the illustrative case with the results that are reported in Figure 7 and Figure 8, with reference values listed in Table 3. These results give a satisfactory positive indication of the functionality of the device and its effectiveness in assisting the extension-flexion motion of the arm for the articulation of the elbow, with the characteristics of motion exercise useful both in rehabilitative, therapeutic applications and in exercises of motion training.

## 5. Conclusions

In this paper, the L-CADEL v2, a lightweight wearable assistive device for elbow exercising and rehabilitation, is presented with its design and operation characteristics. The requirements for elbow exercising, both from technical and medical points of view, are reviewed and discussed to improve previous designs by considering not only the technical performance of the device but also the comfort of a patient and ease of use. A novel design is introduced with its main features and constructive details, and a prototype is built at the LARM2 laboratory in Rome. Experimental results validate the feasibility of the proposed assistive device and show its operational performance in terms of angular motion, acceleration, and power consumption. Thus, the L-CADEL v2 provides a solution for a lightweight, low-cost, portable, and easy-to-use device for assistive and rehabilitation exercising of the elbow. 

## 6. Patents

1. Giuseppe Carbone, Marco Ceccarelli, *Cable-based system for motion assistance*, Patent no. IT. 102016000038975-06/11/2018, Italy.
2. Marco Ceccarelli, Lucia Ferrara, Victor Petuya, *Device for elbow rehabilitation*, Patent no. IT. 102017000083887–29/10/2019, Italy
3. Marco Ceccarelli, Matteo Russo, *Device for motion assistance of ankle*, n. 102020000002863, 13/02/2020, Italy, patent pending.
4. Marco Ceccarelli, Matteo Bottin, Giulio Rosati, *Portable cable-driven exoskeleton for elbow motion assistance*, n. 102020000004885, 9/3/2020, Italy, patent pending.
5. Marco Ceccarelli, Matteo Russo, Monica Urizar Arana, Mykhailo Riabtsev, Axel Fort, Mohamed Amine Laribi, *Portable device for motion assistance of the elbow*, n. 102021000013229, 20/5/2021, Italy, patent pending.

## Figures and Tables

**Table 1:** Existing upper-limb rehabilitation/exercising exoskeleton robots.

Design	Joint	DoF	Actuators	Power Transmission
ARMin [33]	shoulder, elbow, wrist	7	DC Brushed Motors, Maxon series RE	cable, gear
CAREX [34]	shoulder, elbow	4	Brushless Motors, Maxon EC 45	cable
EXO-7 [35]	shoulder, elbow, wrist	7	DC Brushed Motors, Maxon	cable
L-EXOS [36]	shoulder, elbow	4	AC Motors, VERNITRON 3730V-115	cable, gear
IntelliArm [37]	shoulder, elbow, wrist, hand	8 + (2)	Electric motors	cable, gear
MEDARM [38]	shoulder, elbow	6	Electric motors	cable, gear
MGA [39]	shoulder, elbow	5	DC Brushless Motors	gear
SUEFUL-7 [40]	shoulder, elbow, wrist	7 + (1)	Harmonic Drive series RH/RHS	cable, gear
MariBot [41]	shoulder, elbow	3 + (2)	Direct drive pulley-motor system	cable

**Table 2:** Size of the design parameters in Figure 3.

Parameter	h_U_	b_U_	s_Umax_	s_Umin_	R_U_	h_L_	b_L_	s_L_	R_L_
Dimension (mm)	99	144	50	30	50	60	60	20	20

**Table 3:** Experimental test results.

	Elbow Angle, Min–Max (deg)	Acceleration Magnitude Min–Max (m/s^2^)	Power Consumption (J)	EMG Response Min–Max (Pulse)
**Free-load**	49.19–90.79	0.073–0.109	37.68	67–176
**With load of 5N**	61.53–110.15	0.102–0.107	37.29	136–201

## Data Availability

Not applicable.

## Appendix A. Protocol for Experimental Activity

1. A test operator sets up the test configuration and system (with/without additional load).
2. A test operator switches on the system (device, PC, data logging program).
3. A test operator checks the sensors, the servomotors, and Arduino.
4. A test operator informs the volunteer of the test and records the filled-in form.
5. A test operator asks the volunteer to sit on a chair with a specific testing configuration.
6. A test operator provides the volunteer with the alcohol solution for the skin treatment.
7. A test operator asks the volunteer to wipe the skin of the shoulder with the alcohol solution.
8. A test operator asks the volunteer to put the pads for the EMG sensor electrodes on his/her arm.
9. A test operator asks the volunteer to connect the electrodes of the EMG sensor to the pads.
10. A test operator asks the volunteer to wear the upper module.
11. A test operator pumps the inflatable cuff.
12. A test operator asks the volunteer to wear the lower module.
13. A test operator asks the volunteer to connect the modules with the cables.
14. A test operator turns on the power of the prototype.
15. A test operator asks the volunteer to stand up and adjust the cables, so the angle between his/her arm and forearm will be 90°.
16. A test operator asks the volunteer to adjust the cable tension so that the cables would support the weight of the forearm.
17. A test operator starts collecting data.
18. A test operator starts the experiment.
19. A test operator finishes collecting data after three full cycles of the motions.
20. A test operator switches off the system.
21. A test operator asks the volunteer to take off the prototype.
22. A test operator provides the volunteer with the comments questionnaire.
23. A test operator undertakes post processes, and checks acquired data.
